# Artificial intelligence, autism care, and health equity: a public health narrative review

**DOI:** 10.3389/fpubh.2026.1898818

**Published:** 2026-07-13

**Authors:** Xugao Han, Lingyan Weng, Houxi Xu

**Affiliations:** 1 School of Medical Humanities, Nanjing University of Chinese Medicine, Nanjing, China; 2 School of Tourism Management, Nanjing Institute of Tourism and Hospitality, Nanjing, China; 3 Key Laboratory of Acupuncture and Medicine Research of Ministry of Education, Nanjing University of Chinese Medicine, Nanjing, China

**Keywords:** access to care, artificial intelligence, autism spectrum disorder, digital health, health equity, machine learning, public health, screening

## Abstract

Autism spectrum disorder (ASD) is a common, lifelong neurodevelopmental condition whose recorded prevalence, diagnostic delays, and uneven distribution of specialist services create a growing public health challenge. Conventional screening and diagnostic pathways depend heavily on scarce specialist expertise, contributing to long waiting times and unequal access across income settings, regions, sex, ethnicity, language, and social position. This narrative review synthesises current applications of artificial intelligence (AI) and machine learning in autism screening, diagnostic support, intervention, and longitudinal monitoring, and reframes the evidence through a public health and health equity lens. We argue that AI’s most important contribution to autism care is unlikely to lie in marginal improvements in classification accuracy alone. Rather, its potential value lies in expanding access, supporting task-sharing, shortening diagnostic pathways, enabling population-oriented screening, and reaching under-recognised groups such as girls and women, adults, ethnic and linguistic minorities, and populations in low-resource settings. At the same time, AI may create an equity paradox: technologies intended to reduce disparities may reproduce or amplify them if they are trained on non-representative data, deployed across a digital divide, or governed without adequate attention to privacy, accountability, and community trust. Whether AI narrows or widens autism-related health inequalities will depend on choices about data diversity, low-resource design, co-design with autistic communities, equity-sensitive evaluation, clinical integration, and proportionate regulation.

## 1 Introduction

### 1.1 Autism as a public health priority

Autism spectrum disorder is a heterogeneous neurodevelopmental condition characterised by differences in social communication and interaction, together with restricted, repetitive patterns of behaviour and interests. Once considered rare, autism is now recognised as one of the most prevalent neurodevelopmental conditions worldwide. The Global Burden of Disease (GBD) Study 2021 estimated that approximately one in every 127 people, or 61.8 million individuals, was on the autism spectrum globally, and ranked autism among the leading contributors to non-fatal health burden in people younger than 20 years (1). A comprehensive systematic review and meta-analysis similarly documented substantial and rising recorded prevalence, with regional variation reflecting differences in ascertainment as well as possible differences in occurrence (2). The GBD analysis of disorders affecting the nervous system further reinforced the population-level importance of autism by listing it among the 10 conditions contributing the greatest age-standardised burden in that broad category (3).

The burden of autism extends beyond diagnosis itself. It includes lifelong support needs, the emotional and economic demands placed on families and caregivers, and pressure on education, health, and social-care systems. Because much of the non-fatal burden is concentrated in childhood and adolescence, timely identification and support are especially important for developmental trajectories and long-term participation (1). Autism is therefore not only a clinical or educational issue, but also a public health priority: it is common, lifelong, associated with substantial population burden, and shaped by preventable inequities in access to detection and care.

Two features are central to a public health account of autism. First, increases in recorded prevalence are driven partly by diagnostic change, broadened diagnostic criteria, greater awareness, and improved ascertainment rather than by changes in underlying incidence alone (4, 5). This means that measured prevalence is strongly influenced by a health system’s ability to identify autistic people. Where detection capacity is limited, including rural areas, low-income communities, and many low- and middle-income countries (LMICs), recorded prevalence may underestimate true need. Second, access to early diagnosis and support is socially patterned. Girls, women, ethnic and linguistic minorities, rural residents, adults, and families with fewer resources are more likely to be identified late or missed altogether (6, 7). Any technology proposed for autism care must therefore be evaluated not only by whether it works on average, but also by whether it reaches and benefits groups that current systems underserve.

### 1.2 Bottlenecks in current screening and diagnostic pathways

Despite broad agreement that early identification and timely developmental evaluation can improve access to support, the pathway from first concern to confirmed diagnosis remains slow, resource-intensive, and unequal (8). Diagnosis commonly relies on specialist-administered behavioural observation, structured interviews, and multidisciplinary assessment. These approaches can be clinically valuable, but they require trained personnel whose availability is limited even in high-income health systems. Reviews of early diagnosis emphasise both the developmental advantages of acting early and the practical difficulties of providing timely, accurate assessment at scale (9). Quality-improvement work in primary care has similarly shown that, without deliberate pathway redesign, only a minority of referred children receive developmental evaluation within clinically meaningful timeframes (10).

These bottlenecks are more severe across the global income gradient. A review of reviews on autism in LMICs identified pervasive barriers, including stigma, limited public and professional awareness, shortages of culturally and linguistically appropriate tools, and specialist-dependent diagnostic models that cannot scale in settings with very few relevant clinicians (11). These barriers are also visible in primary clinical data. In a six-year cohort at a Nigerian paediatric neurology clinic, autistic disorder accounted for 2.3% of new presentations, yet although parents first noticed atypical development at a mean age of 22.5 months, the mean age at diagnosis was 44.7 months—a delay of roughly 2 years that the authors attributed in part to limited awareness and specialist-dependent referral pathways (12). Regional GBD analyses, including those for the Association of Southeast Asian Nations, document both increasing recorded prevalence and sparse epidemiological data, suggesting large pools of unmet and undetected need (4). The result is a detection gap and a treatment gap: many autistic children and adults are identified late or never, and many who are identified still do not receive timely, appropriate support.

### 1.3 Why AI requires a public health evaluation

It is in this context that AI has attracted attention. The appeal is not merely technical. AI-based systems can analyse behavioural and physiological signals, such as gaze, facial expression, voice, language, movement, and sensor-derived patterns, and can be embedded in tablets, smartphones, wearables, telehealth systems, and clinical decision-support tools. In principle, such tools could be deployed repeatedly, remotely, and at low marginal cost. They could help identify children who need specialist assessment, support non-specialist providers, personalise intervention, and monitor change over time.

However, the public health question is different from the engineering question. A model that performs well in a curated research sample may not improve health outcomes if it is inaccessible, unaffordable, unacceptable, biased, or disconnected from services. Conversely, a tool with modest diagnostic performance may have substantial public health value if it safely triages need, reduces waiting times, and expands access in settings where no specialist pathway currently exists. For this reason, AI in autism should be assessed not only by accuracy, sensitivity, specificity, or area under the curve, but also by reach, equity, implementation feasibility, acceptability, privacy, and integration into care pathways.

The concern that interventions intended to reduce disparities can instead widen them is well established in public health. Frohlich and Potvin termed this an “inequality paradox”: population-level interventions may enlarge health inequalities because more advantaged groups are better able to convert newly available resources into health gains (13). This restates, in contemporary form, Tudor Hart’s inverse care law, whereby the availability of good care tends to vary inversely with the need of the population served (14). In data-driven systems, the paradox acquires a further mechanism. Models trained on non-representative data can encode and then scale existing disparities, as shown when a widely used healthcare risk-prediction algorithm systematically under-identified Black patients with complex needs because it used past healthcare expenditure as a proxy for illness (15). More broadly, scholarship on the social embedding of technology has shown how automated and algorithmic systems can reproduce racial and structural disadvantage even when designed without discriminatory intent (16, 17). The equity paradox examined in this review is therefore not a new principle but an application of this established body of work to a specific and under-examined setting—AI-enabled autism care—together with an analysis of the particular pathways through which it may operate there.

Against this risk, emerging technical approaches are being developed to reconcile the competing demands of data diversity and privacy. Privacy-preserving and decentralised methods such as federated learning, which allow models to be trained across institutions without centralising sensitive data, have been proposed as a way to assemble the representative datasets that equitable AI requires while protecting privacy and enabling more equitable institutional participation; we return to these approaches in Section 6.1 (18).

### 1.4 Aim and contribution of this review

A large and rapidly expanding literature now addresses AI in autism. Existing reviews have described the transition from traditional approaches to AI (19), proposed methodological innovation strategies such as multimodal data fusion, federated learning, and digital twins (20), and catalogued AI diagnostic methods using face, voice, and text analysis (21). Much of this literature is organised around technical performance. Less attention has been paid to the population-level questions that define public health: who is reached, who is missed, whether disparities are reduced, and what forms of governance are needed for safe and equitable implementation.

This narrative review addresses that gap. We first describe how AI is being applied across the autism care continuum, including early screening, diagnostic support, intervention, and longitudinal monitoring. We then examine the public health implications of these applications, with attention to access, task-sharing, diagnostic delay, cost, and under-recognised populations. Finally, we analyse the risks and limitations that create an equity paradox, and outline future directions for an equity-oriented AI agenda in autism care. In doing so, the review’s conceptual contribution lies not in the equity paradox itself, which is well established, but in mapping it systematically across the autism care continuum and in identifying mechanisms specific to autism—such as the interaction between social camouflaging in autistic girls and women and male-dominated training data, and the misreading of culturally variable gaze, expression, and interaction styles—through which AI may widen rather than narrow inequities.

## 2 Scope, literature identification, and narrative synthesis approach

This article is a narrative review rather than a systematic review. It does not aim to provide exhaustive coverage, formal risk-of-bias assessment, or quantitative synthesis. Instead, it offers a thematically organised synthesis of current evidence and policy-relevant issues at the intersection of AI, autism care, public health, and health equity. A structured literature identification process was used to improve transparency, while retaining the interpretive flexibility appropriate to a narrative public health review.

The primary information source was PubMed/MEDLINE. Searches were conducted up to 2 June 2026. We prioritised publications from January 2021 to June 2026 because AI methods and digital health applications are evolving rapidly, while retaining selected earlier sources when they remained foundational for a concept or burden estimate. PubMed was used as the primary database because the review focuses on clinical, public health, and digital health implications rather than a comprehensive catalogue of engineering methods. We recognise that this choice may have omitted relevant technical work indexed primarily in IEEE Xplore, ACM Digital Library, arXiv, or other computer-science databases. In addition, foundational theoretical and policy sources that fall outside the database search—including books and earlier conceptual work not indexed in PubMed, such as established health-equity frameworks—were identified through citation snowballing from key references, so that the review’s conceptual grounding did not depend on the primary search window alone.

Search terms were organised into three conceptual blocks. The population block included “autism spectrum disorder,” “autism,” “autistic,” “ASD,” and “neurodevelopmental disorder.” The technology block included “artificial intelligence,” “machine learning,” “deep learning,” “computer vision,” “natural language processing,” “large language model,” “digital phenotyping,” “robot,” and “wearable.” The application and public health block included “screening,” “diagnosis,” “early detection,” “intervention,” “telehealth,” “digital health,” “access,” “health equity,” “disparities,” “low- and middle-income countries,” “algorithmic bias,” “fairness,” “federated learning,” and “regulation.” Representative queries included combinations such as “artificial intelligence AND autism spectrum disorder AND review,” “machine learning AND autism AND screening AND early detection,” “autism AND telehealth AND digital health,” and “artificial intelligence AND autism AND (health equity OR disparities OR access).”

Records were screened for relevance to four organising themes: AI applications across the autism care continuum; public health implications for access and scalability; equity implications for under-recognised populations; and ethical, governance, and regulatory challenges. Records were included if they addressed artificial intelligence or machine learning in relation to autism across these themes and were published in English; we excluded records that were purely technical without clinical, public health, or implementation relevance, that did not concern autism, or for which only an abstract was available. Several hundred records were screened at the title and abstract level, from which the studies discussed here were selected on the basis of relevance, methodological contribution, and coverage of under-represented populations and settings. We prioritised systematic reviews, meta-analyses, large or prospective primary studies, real-world implementation studies, and work that explicitly addressed equity, LMICs, sex and gender differences, cultural and linguistic diversity, or algorithmic fairness. Because autism-specific evidence on fairness, regulation, and privacy-preserving learning remains limited, selected methodological and policy references were drawn from the broader health-AI literature. Where a key empirical claim risked resting only on secondary syntheses, we sought to anchor it additionally in primary observational or trial evidence; reviews of reviews were retained for breadth of coverage but, wherever possible, were supplemented with primary studies. Consistent with a narrative rather than systematic design, selection was purposive and interpretive: we did not apply a formal study-selection protocol, quantitative synthesis, or risk-of-bias assessment, and we describe these procedures to make our approach transparent rather than to imply the reproducibility of a systematic review. The synthesis is therefore interpretive and integrative rather than exhaustive.

## 3 AI applications across the autism care continuum

**Figure 1:**
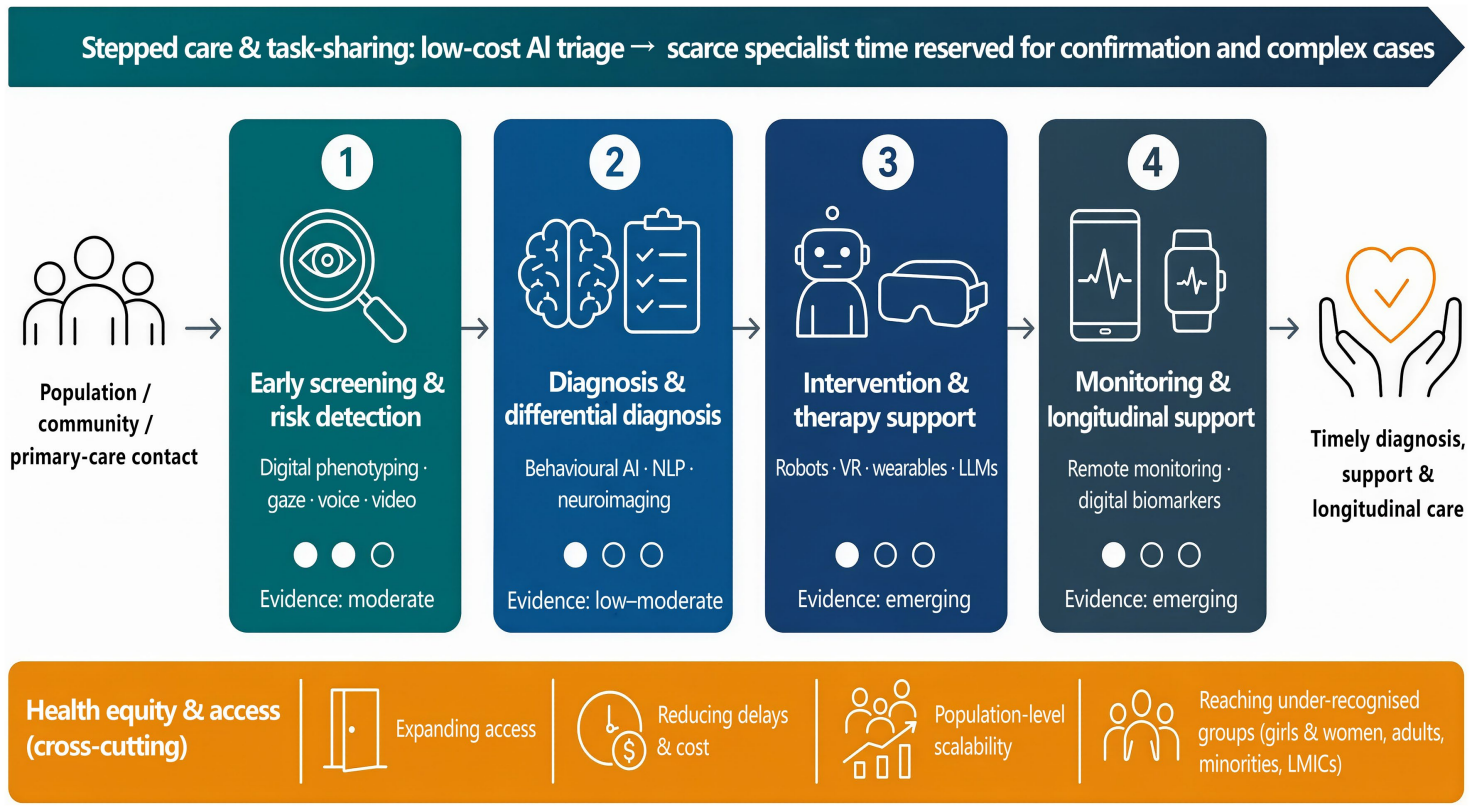
Artificial intelligence across the autism care continuum and its public-health leverage points. Four sequential domains, screening, diagnosis, intervention, and monitoring, are shown within a stepped-care pathway from population-level contact to specialist support, with health equity and access represented as a dimension cutting across all stages.

AI applications in autism can be grouped across the care continuum: early screening and risk detection, diagnostic support, intervention and therapy support, and longitudinal monitoring. Figure 1 provides a conceptual overview of how AI maps onto the autism care continuum, with health equity as a cross-cutting dimension. These domains differ markedly in evidence maturity, implementation readiness, and equity implications. Table 1 summarises the main application areas, types of AI methods, current evidence maturity, public health relevance, and key equity concerns.

**Table 1:** AI applications across the autism care continuum: evidence maturity, public health relevance, and equity concerns.

Care-continuum domain	AI modalities and examples	Evidence maturity	Public health relevance	Main equity concerns
Early screening and risk detection	Digital behavioural phenotyping; computer vision; eye-tracking; facial, vocal, motor, and task-performance analysis; smartphone- or tablet-based tools.	Moderate but heterogeneous. Evidence includes reviews and prospective implementation-oriented studies, but many studies remain small or demographically narrow.	High. Screening is the population-facing entry point and could support earlier detection, triage, and task-sharing in primary care or community settings.	Risk of reduced performance in under-represented groups; cultural variation in gaze, expression, and language; uneven access to devices and connectivity.
Diagnosis and differential diagnosis	AI analysis of social behaviour, facial features, language, clinical notes, neuroimaging, and multimodal data.	Low to moderate. Some meta-analytic support exists for behavioural analysis, while neuroimaging and language-based approaches remain largely research-oriented.	Variable. Behavioural and language tools may support clinicians, but MRI-based models are less scalable for underserved settings.	Training datasets often over-represent high-income, male, white, and English-speaking samples; risk of missing girls, women, adults, and culturally diverse presentations.
Intervention and therapy support	Socially assistive robots; virtual reality; game-based tools; wearables; adaptive software; emerging generative AI and large language model applications.	Emerging. RCT evidence is strongest for selected robot-assisted approaches, while many interventions have small samples, short follow-up, and heterogeneous outcomes.	Moderate. Tools may extend therapy into homes and schools, increase engagement, and reduce dependence on specialist centres if affordable and acceptable.	Potential mismatch with neurodiversity-informed goals; affordability barriers; limited evidence on generalisation, long-term outcomes, and acceptability to autistic people.
Monitoring and longitudinal support	Remote monitoring platforms; smartphone sensors; wearables; digital phenotyping; environmental and physiological data streams.	Emerging. Platforms are technically feasible, but autism-specific clinical impact and acceptability evidence remain limited.	High if integrated into care pathways. Monitoring may reduce follow-up burden and enable timely support outside specialist clinics.	Privacy risks, consent challenges, data ownership concerns, surveillance burden, and unequal digital literacy or infrastructure.

### 3.1 Early screening and risk detection

Screening is the domain in which AI has the most direct public health relevance because it sits at the point where populations first enter the care pathway. A review of AI methods for autism screening organised the literature around behavioural markers, including gaze behaviour, facial expression, motor movement, voice features, and task performance, and reported a wide range of classifier performance across studies (22). Multimodal approaches often performed better than single-signal approaches, but heterogeneity in samples, stimuli, recording conditions, and analytic pipelines limited generalisability. This variation matters for public health implementation: a model that works well in a controlled research environment may not remain reliable in the more diverse and less controlled conditions of primary care, schools, or community settings.

Eye-tracking and computer-vision studies illustrate both the promise and the limitations of the field. Studies have transformed scan-path patterns elicited by social stimuli into visual representations that can be classified using deep-learning approaches (23). Work in toddlers and preschool children has identified distinct fixation patterns in autistic and typically developing children and has shown that support-vector-machine classifiers can discriminate between groups, while also indicating that performance varies with developmental age (24). Studies using eye movements during viewing of real and animated faces have similarly reported moderate classification performance and suggested that attention to human versus cartoon stimuli may carry diagnostic information (25). These studies demonstrate that gaze-based screening is technically feasible, but their small and often demographically limited samples restrict conclusions about population-level performance.

The most implementation-relevant evidence comes from digital behavioural phenotyping in routine care. A multiclinic prospective study embedded an autism-screening application into well-child visits, using a tablet to elicit behavioural responses, computer vision to quantify digital phenotypes, and machine learning to combine signals into a screening algorithm (26). Importantly, the study reported similar sensitivity across subgroups defined by sex, race, and ethnicity, suggesting that equity-sensitive performance is achievable when diversity is considered during evaluation (26). The significance of this work lies not only in its diagnostic accuracy, but also in its deployment context: it shows how AI screening could be integrated into routine primary-care contact rather than being limited to specialist clinics.

Overall, AI screening tools are promising but not yet ready to be treated as stand-alone diagnostic instruments. Their near-term public health role is more appropriately framed as triage and decision support: helping identify children who should receive further assessment, supporting non-specialist providers, and reducing missed opportunities for referral. Large, prospective, externally validated studies in diverse real-world populations remain essential before such tools can be recommended for broad population-level use.

### 3.2 Diagnosis and differential diagnosis

AI has also been investigated as a support for formal diagnosis and differential diagnosis. A systematic review and meta-analysis of AI for tracking social behaviours and supporting autism diagnosis found that automated analysis of facial and social-interaction features showed clinically meaningful diagnostic performance, particularly when used in unstructured play paradigms and with selected algorithm families (27). Nevertheless, most included studies were small, and the authors emphasised the need for external validation in diverse populations before clinical deployment.

Neuroimaging-based classification represents a more research-oriented strand. Deep-learning approaches applied to structural and resting-state functional MRI have achieved classification accuracies in the high-70s to low-80s percent range, and multimodal fusion of complementary imaging contrasts can outperform single-modality models (28, 29). These studies may contribute to understanding autism heterogeneity and biomarker development. However, their public health relevance is currently limited by reliance on expensive, centre-based imaging infrastructure. Unless imaging-derived insights translate into cheaper and more scalable tools, MRI-based classification is unlikely to reduce access gaps in the settings where unmet need is greatest.

Language-based and natural-language-processing approaches are another emerging area. Adult autism diagnosis is complicated by overlapping psychiatric presentations, subtle phenotypes, and the limitations of instruments developed primarily around childhood and male presentations (30). A study applying a large language model to autism-associated language suggested that such models may identify linguistic features, including echolalia and atypical pronoun use, that are relevant to diagnostic reasoning (31). These approaches remain experimental, but they may eventually complement clinical judgement, especially for groups whose presentations are often missed by conventional tools.

A key tension in the diagnostic literature is that the methods with the most sophisticated data, such as multimodal neuroimaging, are often the least accessible, while the tools with the greatest public health reach, such as smartphone-based behavioural or language analysis, require stronger validation. Future diagnostic-support research should therefore prioritise scalable modalities, disaggregated performance reporting, and prospective evaluation in the populations most affected by delayed or missed diagnosis.

### 3.3 Intervention and therapy support

AI-enabled interventions span socially assistive robots, virtual reality, wearables, game-based systems, adaptive software, and emerging generative AI tools. These modalities differ markedly in evidence maturity, and treating them as a single category risks overstating the readiness of the field. We therefore distinguish three tiers: interventions supported by randomised controlled trials; emerging interventions supported by preliminary or non-randomised evidence; and early-stage, proof-of-concept applications.

The strongest current evidence is for robot-assisted therapy. A recent pair of randomised clinical trials evaluated socially assistive robots targeting joint attention, imitation, and turn-taking in young autistic children (32). An efficacy trial (*n* = 69; mean age 4.4 years) found that 12 biweekly in-clinic sessions produced outcomes equivalent to conventional therapist-delivered treatment, with a significant increase in child engagement, while an effectiveness trial using a simplified, portable set-up suitable for schools or homes achieved equivalent outcomes over only five sessions (32). These are among the more rigorous trials in the field, and the portability of the second protocol is relevant to public health because tools usable outside specialist clinics are more likely to scale. Their implications should nonetheless be interpreted cautiously. Both trials demonstrated equivalence rather than superiority to standard care, so the principal benefits are increased engagement and reduced therapist workload rather than improved clinical outcomes; the samples were modest and confined to preschool-aged children, leaving effects in older children, adolescents, and adults untested; the intervention doses were short, with limited evidence on the maintenance or generalisation of skills; and both trials originated from a single research programme, so independent replication in other cultural, linguistic, and resource settings is still required. Robot-assisted therapy is therefore promising but not yet a mature, broadly validated intervention.

Evidence from this tier also counsels against assuming that technological delivery is interchangeable with human or animal interaction. A randomised trial comparing dog-assisted and robot-dog-assisted therapy found greater gains in emotional attunement and regulation in the living-dog condition (33), a reminder that substituting a device for a relational partner can change what an intervention achieves.

A second tier of emerging interventions—virtual reality, wearables, game-based systems, and adaptive software—rests on preliminary and largely non-randomised evidence. Systematic reviews of AI interventions for eye contact and for social or psychological enhancement report improvements in selected outcomes such as joint attention, social communication, emotion recognition, and engagement, but they consistently identify small samples, short intervention durations, inconsistent outcome definitions, and little evidence on generalisation or long-term effects (34, 35). These tools are plausible but not yet supported by the evidence required for confident implementation.

A third tier comprises early-stage, proof-of-concept applications, most notably generative AI and large language models. At present, this literature is largely conceptual: commentaries have proposed roles in personalised, context-aware communication support, social coaching, and educational assistance (36), but controlled empirical evaluation in autistic populations is largely absent. Reliability, safety, misinformation risk, data privacy, and dependence on human oversight are particularly salient when such tools are used with children or other vulnerable users, and these applications are best regarded as hypotheses to be tested rather than as interventions ready for deployment.

Intervention research also raises a normative issue. Some AI interventions aim to increase behaviours such as eye contact or reduce visible autistic behaviours. Such goals should not be assumed to be universally desirable. A neurodiversity-informed approach should evaluate whether interventions support communication, autonomy, participation, well-being, and autistic flourishing, rather than simply promoting behavioural normalisation. Co-design with autistic people and families is therefore not only ethically important, but also necessary for acceptability and real-world relevance.

### 3.4 Monitoring and longitudinal support

AI-based monitoring has received less attention than screening or diagnosis, but it may become important for long-term support. Remote-monitoring platforms can collect longitudinal data from smartphones, wearables, and connected devices, generating digital biomarkers across behavioural, environmental, and physiological domains. Platforms such as RADAR-base have been used across mental and physical health conditions, including autism-related contexts, to support remote monitoring and personalised follow-up (37). In principle, such tools could track response to intervention, identify changes in stress or functioning, and reduce the burden of repeated in-person specialist visits.

However, acceptability cannot be assumed. A mixed-methods study of remote daily-living support for neurodivergent young adults found that although remote support could increase accessibility and choice, service users were more cautious than providers and raised concerns about miscommunication and reduced social or emotional connection (38). For public health implementation, this finding is important: a tool that extends reach but is experienced as inferior or intrusive may improve nominal access without improving outcomes. Longitudinal AI support should therefore be evaluated for acceptability, burden, privacy, and integration with human care, not only for technical feasibility.

## 4 Public health implications for equitable autism care

### 4.1 Expanding access through task-sharing and stepped care

The strongest public health rationale for AI in autism is its potential to extend detection and support beyond specialist centres. In many settings, the main constraint is not that specialists cannot diagnose autism accurately, but that too few specialists are available relative to need. AI tools may help shift autism care toward stepped models in which low-cost, standardised screening is delivered in primary care, schools, community programmes, or homes, while scarce specialist time is reserved for confirmation, complex cases, and care planning.

This role aligns with task-sharing approaches recommended in global mental health and autism care. Reviews of autism services in LMICs emphasise that universal coverage will require culturally adapted tools, community participation, frontline workforce development, and technological innovation (11). That such task-sharing is feasible is supported by primary trial evidence. In a single-blind randomised controlled trial conducted in India and Pakistan, a parent-mediated intervention delivered by non-specialist health workers—rather than by scarce specialists—produced significant improvements in parent–child communication relative to treatment as usual, demonstrating that structured autism support can be delivered by trained non-specialists in low-resource settings (39). AI should be understood as a capacity-extending tool rather than a replacement for clinicians. A well-designed screening application could guide community health workers, primary-care nurses, teachers, or caregivers through an initial assessment, while creating a structured pathway for referral and supervision. Such a model is more plausible and more equitable than visions of fully automated diagnosis.

### 4.2 Reducing diagnostic delays and pathway costs

A second public health contribution is the potential to reduce diagnostic delay and the cost of assessment. Conventional pathways often involve multiple appointments, specialist observation, and long waiting lists. Even modest reductions in the interval between first concern, developmental evaluation, and intervention may be meaningful because early developmental periods are particularly important for access to support (8, 9). AI can contribute by triaging referrals, automating selected observations, supporting non-specialists, and helping prioritise children who require comprehensive assessment.

The economic logic is distinct from the accuracy logic. Low-cost screening at scale changes the allocation of specialist resources. Instead of relying on specialist assessment as the first point of entry, health systems could use AI-supported screening to identify those at a higher likelihood of autism or developmental need and then allocate more intensive assessment accordingly. This approach will only reduce inequity if downstream services exist. Screening without available diagnostic confirmation, intervention, or family support merely relocates the bottleneck.

### 4.3 Population-level screening and under-recognised groups

AI could also support a shift from reactive referral-based identification toward proactive population-oriented developmental surveillance. Universal developmental screening is widely endorsed but inconsistently implemented. Conventional caregiver questionnaires can perform unevenly in routine care and may miss children whose presentations differ from the populations on which the tools were developed. Digital behavioural phenotyping offers a potential objective complement, particularly if it can be embedded in routine well-child visits or community health contacts (26). One of the most important equity opportunities is reaching groups that have been under-recognised in conventional diagnostic pathways (6, 7). Autism in girls and women is often diagnosed later and less consistently than in boys and men. Systematic reviews of autism in women and camouflaging have described diagnostic inequities related to masking, less externalising presentations, and reduced sensitivity of standard instruments (40, 41). Misdiagnosis is also a concern; autistic girls and women may be misdiagnosed with borderline personality disorder, with downstream consequences for support and care (42). AI-based markers of behaviour, gaze, language, or longitudinal patterns could, in principle, detect presentations that conventional tools miss. However, this opportunity depends entirely on whether these groups are adequately represented in training and validation data.

### 4.4 Implementation conditions for public health benefit

Public health value should not be inferred from model performance alone. To improve equity, AI tools must be affordable, usable by non-specialists, culturally and linguistically adapted, accessible to people with limited connectivity or digital literacy, and embedded in functioning care pathways. A large survey of parents and rehabilitation therapists in two Chinese provinces found low current use of digital health services for autism, but strong interest in smartphone-based platforms; it also identified cost, additional equipment, usability, and regional disparities as key barriers (43). This illustrates the difference between demand and equitable uptake. Digital tools may be welcomed, but their benefits will not reach underserved families unless affordability and usability are treated as core design requirements.

Implementation research should therefore measure outcomes that matter for public health: time to assessment, referral completion, age at diagnosis, family burden, intervention uptake, equity of access across demographic groups, and acceptability to autistic people and families. AI-enabled screening should be evaluated as part of a pathway rather than as an isolated test, consistent with broader guidance on the early-stage clinical evaluation and reporting of AI-based decision-support systems (44). These outcomes are as important as sensitivity or specificity when evaluating whether AI improves autism care at scale.

## 5 Risks, limitations, and the equity paradox

**Figure 2:**
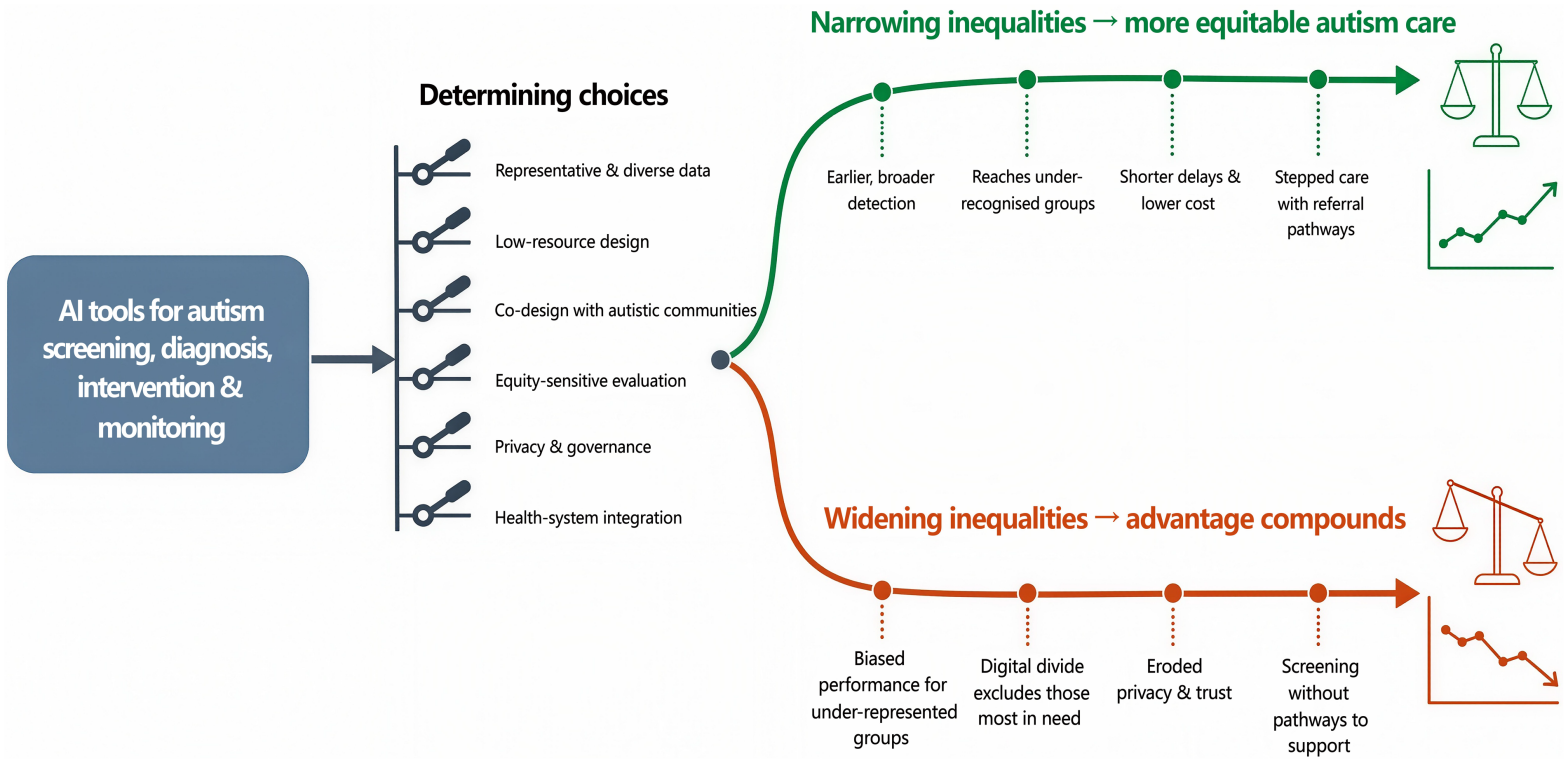
The equity paradox as it applies to AI-enabled autism care—an applied instance of a long-recognised pattern in public health and algorithmic systems rather than a novel principle. The same AI tools can follow two divergent trajectories—narrowing or widening autism-related health inequalities—depending on deliberate choices about representative data, low-resource design, co-design, equity-sensitive evaluation, privacy and governance, and health-system integration.

The same technologies that might expand access can also reproduce or amplify existing disparities. As noted in the Introduction, this risk is not unique to AI: it is the algorithmic expression of a long-recognised pattern in which interventions intended to reduce inequity may instead entrench it (13–17). We use the term “equity paradox” to denote this dynamic as it applies to AI-enabled autism care, and our aim in this section is to specify the mechanisms through which it operates in this domain rather than to restate the general principle. Several are especially important. Figure 2 summarises this equity paradox and the choices that determine which trajectory prevails.

### 5.1 Algorithmic bias and non-representative data

Machine-learning systems learn from the data on which they are trained. If datasets over-represent high-income, white, male, English-speaking children, models are likely to perform best for those groups and less well for girls, women, adults, ethnic and linguistic minorities, and LMIC populations. This is not only a technical limitation; it is a public health risk. In mental health, studies of machine learning for depression prediction have shown that standard models can behave unfairly across populations and that fairness must be examined during model selection and evaluation (45). Reviews of algorithmic fairness in laboratory medicine similarly warn that real-world data reflect societal biases and that careless machine-learning deployment can perpetuate or amplify disparities (46). Empirical oncology work has demonstrated how models can reproduce and magnify racial disparities present in registry data (47).

The autism-specific implications are direct. A gaze or facial-expression model trained mainly on boys may under-detect autistic girls. A language model trained on English-speaking children may not generalise to other languages or cultural communication norms. A behavioural model calibrated on one population may misinterpret culturally normative eye contact, facial expressivity, or interaction style in another. Because AI outputs may appear objective, biased results could be harder for families or clinicians to challenge than subjective judgments.

Feedback loops are another concern. If biased tools influence diagnostic labels, those labels may enter future datasets, reinforcing the initial bias. Social camouflaging, particularly among girls and women, poses a specific challenge: models trained on overt presentations may be less able to detect masked or subtle presentations (41). Equity must therefore be engineered from the beginning through representative recruitment, inclusive labelling practices, disaggregated validation, and active bias mitigation.

### 5.2 The digital divide

Even a fair algorithm cannot help people who cannot access it. AI-enabled autism tools often assume smartphones, tablets, reliable electricity, connectivity, private space, and digital literacy. These resources are unevenly distributed within and between countries. The Chinese stakeholder survey showed that cost, equipment needs, usability, and regional differences affected digital health service use and demand (43). If adoption follows existing social advantage, digital tools may widen gaps by benefiting families who already have better access to care. Inclusive design, offline functionality, low-cost deployment, caregiver support, and infrastructure investment are therefore essential components of equitable implementation.

### 5.3 Privacy, consent, and data governance

AI autism tools often depend on sensitive data: video of children’s faces and gaze, voice recordings, language samples, behavioural observations, physiological signals, and sometimes neuroimaging. These data raise acute concerns about privacy, security, consent, future reuse, data ownership, and potential stigma if information is breached or misused. Children may not be able to provide fully informed consent, and families may feel pressure to accept digital tools if access to services depends on them. These concerns are heightened in low-resource settings with weaker data-protection infrastructure, where the need for scalable tools may also be greatest. Technical privacy protections are important, but governance questions are equally central: who controls the data, who benefits from model development, and how families and autistic communities can hold developers and health systems accountable.

### 5.4 Validation, generalisability, and clinical translation

A consistent limitation across AI applications in autism is methodological immaturity. Reviews of screening, diagnosis, and intervention repeatedly note small samples, narrow demographic composition, heterogeneous outcome measures, short follow-up, and limited external validation (22, 27, 34, 35). High accuracy in one dataset does not guarantee safe or equitable performance in another setting. For public health implementation, generalisability is a primary requirement, not a secondary technical concern. Tools intended for population-level use should undergo prospective, multi-site validation in the settings and populations where they will be deployed, with transparent reporting of workflow, human-AI interaction, error modes, and implementation context (44).

Clinical translation also requires clarity about the role. AI tools should not be framed as replacing diagnostic judgment unless evidence supports that use. More realistic near-term roles include triage, documentation support, risk stratification, intervention personalisation, and longitudinal monitoring. Each role carries different requirements for accuracy, explainability, oversight, and accountability.

### 5.5 Ethical and regulatory gaps

The governance environment for medical AI remains underdeveloped relative to the pace of innovation, despite growing international guidance on ethics, human rights, transparency, accountability, and equity in AI for health (48). Reviews of clinical AI implementation describe uncertainties around approval, monitoring, reimbursement, accountability, and the regulation of adaptive or generative systems (49). Analyses of AI and machine-learning regulation emphasise safety, security, bias, accountability, and trust as central concerns (50). Generative AI introduces additional risks. A study of large language model vulnerabilities showed that such systems could be converted into convincing health-disinformation chatbots, including the propagation of discredited claims such as a vaccine-autism link (51). In autism care, where misinformation and stigma already cause harm, unregulated generative AI advice could undermine families’ trust and decision-making.

## 6 Future directions

An equity-oriented AI agenda for autism should shift priorities from maximising accuracy on convenient datasets toward demonstrating real-world, population-level benefit. The challenges this agenda must address—data diversity, algorithmic bias, limited clinical validation, and the need for transparent, explainable, and clinician-trustworthy systems—mirror those identified across the broader field of artificial intelligence for mental health, where they are similarly recognised as decisive for equitable implementation (52). Several directions follow.

### 6.1 Diverse, representative, and privacy-preserving data

The field needs diverse and representative data. Datasets should include girls and women, adults, ethnic and linguistic minorities, rural communities, and LMIC populations. Privacy-preserving collaboration will be necessary because autism-related data are sensitive and often siloed. Federated learning allows models to be trained across multiple sites without centralising raw data; demonstrations in health research suggest that federated models with differential privacy can approach centralised analyses while improving privacy protection (53). A recent systematic review of the ethics of federated learning in healthcare reinforces, however, that its contribution extends beyond privacy: responsible deployment also requires attention to algorithmic fairness, governance, and equitable access to the computational resources that institutional participation demands (18). Framed this way, federated learning is best understood not as a technical solution to inequity but as one component of an equity-oriented data strategy. Such approaches could allow minority-serving and LMIC institutions to contribute to and benefit from model development on more equitable terms.

### 6.2 Low-resource and real-world implementation design

AI tools should be designed for low-resource and real-world settings from the start. This means lightweight models that run on inexpensive devices, offline capability, simple interfaces for non-specialists and caregivers, culturally adapted content, and minimal equipment requirements. The portable robot-assisted therapy trials and the primary-care digital phenotyping study provide useful examples of implementation-oriented design (26, 32). Explainability is an increasingly important component of such implementation-oriented design, because tools that are to be used by non-specialists and trusted by families must be interpretable as well as accurate. Recent work illustrates this direction by integrating an interpretable, SHAP-based model into a deployable web application for ASD detection (54). However, the very high accuracies often reported in this literature commonly reflect benchmark datasets derived from the items of screening instruments themselves, rather than independent diagnostic validation; such examples should therefore be read as motivating the development of transparent, deployable tools whose performance is then confirmed prospectively in real-world populations, not as evidence of diagnostic accuracy.

### 6.3 Responsible multimodal and large language model integration

Multimodal integration and large language models should be used responsibly. Combining behavioural, linguistic, physiological, environmental, and, where appropriate, neuroimaging signals may better capture autism heterogeneity than single modalities (20, 28, 29). Large language models may support communication, education, and clinical documentation, but their use must include human oversight, transparency about limitations, safeguards against misinformation, and evidence of safety and benefit in autistic populations (31, 36, 51).

### 6.4 Co-design and neurodiversity-informed outcomes

Co-design with autistic communities should become standard. Tools developed for autistic people without their participation risk optimising the wrong outcomes. Participatory design should include autistic children and adults when appropriate, families, clinicians, educators, and community organisations. Evaluation should consider whether tools support communication, autonomy, participation, and well-being, not only whether they modify observable behaviours.

### 6.5 Equity-sensitive evaluation

Evaluation frameworks must be equity-sensitive and should align with emerging reporting standards for clinical AI evaluation (44). Reporting should include disaggregated performance by sex, gender, race, ethnicity, language, age, socioeconomic status, disability profile, and geography when feasible. Studies should report fairness metrics, access outcomes, acceptability, referral completion, and downstream service uptake. The depression-prediction and laboratory-medicine fairness literatures provide methodological examples for analysing and mitigating bias (45, 46). Without such reporting, the field cannot determine whether AI is closing or widening gaps.

### 6.6 Governance and health-system integration

AI must be integrated into health systems and governed proportionately. Regulatory and governance frameworks should balance innovation with safety, privacy, transparency, accountability, human oversight, equity, and post-deployment monitoring (48–50). Health systems should define how AI outputs are reviewed, how families can contest results, how data are protected, and how screening links to diagnostic and intervention capacity. Screening that identifies need without a route to support is not a public health success. The goal should be AI-enabled stepped care aligned with universal health coverage, not isolated tools operating outside service pathways.

## 7 Conclusion

Autism is a common and lifelong public health priority marked by long diagnostic delays and substantial inequities in access to care. AI offers a distinctive opportunity to address these problems by supporting low-cost screening, diagnostic triage, intervention, and longitudinal monitoring across settings that specialist-dependent pathways do not reach. Its greatest public health value lies not in replacing clinicians or maximising accuracy in research datasets, but in changing the distribution of access: who is identified, how quickly, at what cost, and with what pathway to support.

That promise is conditional. AI tools built on non-representative data, deployed across a digital divide, or governed without adequate privacy, accountability, and community involvement may entrench the disparities they claim to reduce. Whether AI narrows or widens inequalities in autism care will depend on deliberate choices about representative data, low-resource design, co-design with autistic communities, equity-sensitive evaluation, and health-system integration. An equity-first public health agenda for AI in autism is both necessary and achievable. Pursued carefully, AI could contribute to more equitable autism care; pursued carelessly, it could become another mechanism through which advantage compounds.
